# Regulation of Stem Cell Properties of Müller Glia by JAK/STAT and MAPK Signaling in the Mammalian Retina

**DOI:** 10.1155/2017/1610691

**Published:** 2017-01-17

**Authors:** Krista M. Beach, Jianbo Wang, Deborah C. Otteson

**Affiliations:** ^1^Department of Basic Sciences, College of Optometry, University of Houston, Houston, TX, USA; ^2^Department of Biology and Biochemistry, University of Houston, Houston, TX, USA

## Abstract

In humans and other mammals, the neural retina does not spontaneously regenerate, and damage to the retina that kills retinal neurons results in permanent blindness. In contrast to embryonic stem cells, induced pluripotent stem cells, and embryonic/fetal retinal stem cells, Müller glia offer an intrinsic cellular source for regenerative strategies in the retina. Müller glia are radial glial cells within the retina that maintain retinal homeostasis, buffer ion flux associated with phototransduction, and form the blood/retinal barrier within the retina proper. In injured or degenerating retinas, Müller glia contribute to gliotic responses and scar formation but also show regenerative capabilities that vary across species. In the mammalian retina, regenerative responses achieved to date remain insufficient for potential clinical applications. Activation of JAK/STAT and MAPK signaling by CNTF, EGF, and FGFs can promote proliferation and modulate the glial/neurogenic switch. However, to achieve clinical relevance, additional intrinsic and extrinsic factors that restrict or promote regenerative responses of Müller glia in the mammalian retina must be identified. This review focuses on Müller glia and Müller glial-derived stem cells in the retina and phylogenetic differences among model vertebrate species and highlights some of the current progress towards understanding the cellular mechanisms regulating their regenerative response.

## 1. Introduction

In humans and other mammals, the retina, like most other regions of the central nervous system (CNS), does not spontaneously regenerate; and damage to the retina or neurodegenerative disease that kills retinal neurons results in permanent blindness. Worldwide, more than 12% of people over the age of 40 have visual impairment or blindness caused by age related macular degeneration and glaucoma, two of the neurodegenerative diseases affecting the retina [1, 2]. As life expectancy continues to increase, the increasing prevalence of blinding neurodegenerative disease is reducing productivity and quality of life and imposing significant economic as well as social burdens to individuals, their families, and society. Current therapies can slow progression and delay vision loss but cannot restore lost vision. Consequently, there is increasing interest in identifying approaches for therapeutic retinal regeneration.

A variety of stem cells, including embryonic stem cells (ESCs), induced pluripotent stem cells (iPSCs), mesenchymal stem cells, and fetal-derived neural and retinal stem cells, are currently under investigation for regeneration and subsequent transplantation of retinal neurons (see reviews in [3–10]). With advancements in gene editing using CRISPR/Cas9 technologies and the ability to expand cells in culture prior to differentiation, extrinsic sources such as ESCs and iPSCs are promising for developing strategies to correct preexisting genetic defects in vitro [11]. However, there are potential ethical concerns with the use of ESCs or progenitors from embryonic or fetal tissues, making them less attractive for therapeutic regeneration. Further, extrinsic stem cells will require surgical transplantation and integration of new neurons into existing circuitry. Although the retina is normally an immune privileged tissue, retinal damage and degenerative disease compromise the blood/retinal barrier, allowing ingress of immune cells [12–15]. Therefore, transplantation therapies may also require immunosuppression for long-term viability of the engrafted cells. An intrinsic retinal stem cell would alleviate concerns of integration and immune response and would provide an alternative strategy to complement the use of extrinsic stem cells.

Müller glia are intriguing candidates for intrinsic retinal stem cells. Müller glia are radial glial cells within the retina and are generated from the same lineage as retinal neurons. In the mature retina, Müller glia maintain retinal homeostasis, buffer ion flux associated with phototransduction, and form the blood/retinal barrier within the retina proper. Although they contribute to gliotic responses and scar formation following retinal injury, Müller glia also show regenerative capabilities that vary across species. This review focuses on Müller glia and Müller glial-derived stem cells in the retina and the phylogenetic differences among model vertebrate species and highlights current progress towards understanding and harnessing their regenerative response.

## 2. Retinal Structure and the Origin of Müller Glia

**Figure 1:**
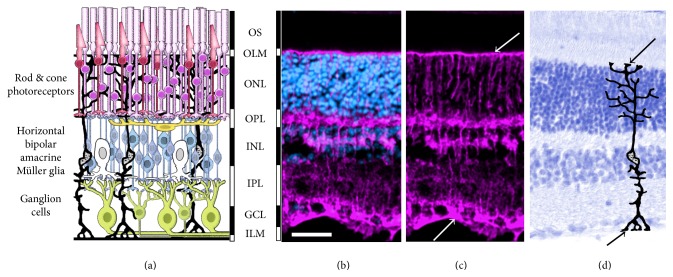
Retinal structure and cellular organization. (a) Diagram shows organization of retinal neurons and Müller glia. The cell bodies of rod (purple) and cone (red) photoreceptors are in the outer nuclear layer (ONL) and the photoreceptor outer segments (OS) contain the photopigments that absorb light. Rod and cone bipolar (blue), horizontal (yellow), and amacrine (white) cells are in the inner nuclear layer (INL), with retinal ganglion cells (green) located in the ganglion cell layer (GCL). Between the nuclear layers are the outer and inner plexiform layers (OPL, IPL) containing the synaptic terminals. Müller glia (black) have cell bodies located in the INL and extend processes throughout the retina. (ILM, OLM). (b, c, d) Photomicrographs of adult mouse retina. (b, c) Müller glia are immunostained for glutamine synthetase (magenta) revealing their radial processes that extend the full thickness of the retina. The Müller glial endfeet form the inner limiting membrane (ILM) and outer limiting membrane (OLM) (arrows). (d) Photomicrograph showing histology of adult mouse retina stained with toluidine blue, showing retinal lamina and overlaid with a diagram of a Müller glial cell. Arrows indicate glial endfeet at ILM and OLM. Scale bar = 50 microns in (b), (c), (d).

The retina is a thin layer of neural tissue located at the posterior pole of the eye. It consists of (a) photoreceptors (rods and cones) that convert light stimuli into neurochemical signals, (b) three major classes of interneurons (horizontal, amacrine, and bipolar cells) that perform initial information processing, (c) Müller glia that perform a multitude of support functions, and (d) projection neurons (retinal ganglion cells) that extend axons through the optic nerve and optic tract to convey the visual image information to higher processing centers within the brain [16–18]. The retinal cells are organized in a highly ordered laminar structure (Figure 1), which allows identification of cell types by position, morphology, and gene expression. The retina is developmentally part of the CNS. Lineage analysis has shown that the multipotent retinal progenitors that make up the embryonic retinal neuroepithelium generate all types of retinal neurons, as well as the Müller glia [16, 19, 20]. Apart from its importance in vision, the neural retina serves as a model system for studying the CNS, as it is the only portion of the central nervous system located outside of the cranium and can be noninvasively imaged and functionally tested in vivo. The process of retinal regeneration recapitulates many aspects of retinal development, with similar patterns of gene expression, cell fate specification, and the order of neurogenesis.

## 3. Müller Glia: Stem Cells for Retinal Regeneration in Fish

The initial evidence for the stem cell characteristics of Müller glia came from research to discover the cellular source of ongoing neurogenesis and regenerative responses in the retinas of fish. The capacity for neurogenesis in the mature retinas of fish appears to be teleologically related to their overall pattern of indeterminate growth and the associated continuous growth of their eyes. Eye growth in fish results in large part from a general expansion/stretching of the retina, leading to decreasing retinal density for most types of retinal neurons within the central retina [21]. There is also ongoing neurogenesis, which occurs at two sites: (1) the circumferential germinal zone (CGZ), where a population of retinal progenitors persists and continually adds concentric rings of new neurons to the retinal margin, and (2) the central retina, where new rods are added continually to the existing photoreceptor mosaic [22]. Neurogenesis of rod photoreceptors in the fish retina begins after hatching and continues throughout life [23–28], with new rod photoreceptors generated from a population of slowly proliferating, PAX6-expressing progenitors within the inner nuclear layer (INL) [29]. These INL progenitors arise from the Müller glia and continue to divide as they migrate to the outer retina to become rod precursors, which subsequently differentiate into rod photoreceptors [26, 28, 29]. Ongoing neurogenesis in the fish retina appears to keep the Müller glia poised to respond to retinal damage by initiating intrinsic neurogenic programs.

Retinal regeneration in fish has been studied for over 50 years [30–34]. A variety of traumatic, surgical, neurotoxic, or phototoxic injuries have been used to induce regenerative neurogenesis, and the outcome is a fully laminated retina, albeit with some relatively minor organizational differences, and a restoration of circuitry and function [34–39]. Although multiple studies had reported clusters of mitotically active cells in the INL following retinal injury [40, 41], the source of the new retinal cells was not identified as Müller glia until 2007 [40–42]. Regardless of the injury paradigm, there appear to be several stages to the retinal response [43]. There is an initial, nonproliferative stage that occurs during the first 2 days after injury, during which Müller glia transiently upregulate expression of the intermediate filament, glial fibrillary acidic protein (GFAP), a key marker of gliosis [44, 45]. The gliotic response of Müller glia is not prominent, and the subsequent, regenerative response in zebrafish begins with an initial, limited proliferation of the Müller glia and generation of a pool of Müller glial-derived progenitors in the INL [46–49]. The Müller glial-derived progenitors continue to proliferate, forming columns of dividing cells that span the retinal layers and, around 7 days after injury, differentiate into retinal neurons [46, 50–52]. Interestingly, the switch from gliosis to proliferation is critical for the regenerative response in zebrafish, as blocking injury-induced proliferation of Müller glia, using 5-fluorouracil or morpholinos against PCNA, enhances the gliotic response, resulting in robust and persistent upregulation of GFAP, and prevents injury-induced neurogenesis [45]. Müller glial-derived progenitors are capable of regenerating all neuronal cell types in an injured retina and restoring visually guided behaviors [34–36, 38, 39, 53–58]. Although early studies suggested that only those subpopulations of neurons that were lost as a result of the initial injury were regenerated [49, 59, 60], recent evidence shows that, even in cases of localized injury to one cell type [e.g., phototoxic injury to photoreceptors or N-methyl-D-aspartate (NMDA) injury to retinal ganglion cells], additional cell types can be produced [48].

## 4. Müller Glia: Gliosis and Injury Response in Mammals and Birds

In warm blooded species, spontaneous regeneration by Müller glia does not occur to any appreciable extent in vivo, leading to questions about the designation of Müller glia as retinal stem cells in these species, particularly in mammals [64]. In the mammalian retina, the primary glial response to retinal injury is gliosis, which is characterized by robust upregulation of GFAP, limited (if any) proliferation, cellular hypertrophy, and formation of glial scars (reviewed in [65, 66]; see also [67–72]). In posthatch birds, there is a slightly more robust response to retinal injury, with gliosis accompanied by a limited neurogenic response that declines with age [64, 73–75].

Evidence for neurogenesis by Müller glia in vivo following acute retinal injury has been demonstrated in posthatch chick, rat, and mouse [62, 64, 71, 73–77] but requires manipulation of various exogenous factors (see below) or overexpression of neurogenic or proneural genes such as* Ascl1a* [78, 79] and* Atoh7* [80, 81]. Intraocular injection of selective neurotoxins that kill either photoreceptors or ganglion/amacrine cells in the adult rodent eye generates large numbers of reactive Müller glia but little proliferation [62, 82]. The proliferative response is enhanced by intraocular injection of a variety of extrinsic growth factors, including CNTF, EGF, FGF1, FGF2, and insulin in posthatch chickens [73–75] and in rodents [62, 71, 83, 84]. However, the overall extent of the neurogenic capacity of mammalian or avian Müller glia is low, even with growth factor stimulation, and, in the mammalian retina, low numbers of neuronal cells are generated and the majority of proliferating progenitors fail to survive in the long term [62]. A better understanding of the mechanisms that promote the proliferative and neurogenic responses is needed before clinically relevant levels of regeneration are achieved in vivo.

## 5. Mechanisms Regulating Gliosis versus Neurogenesis: JAK/STAT versus MAPK Signaling

**Figure 2:**
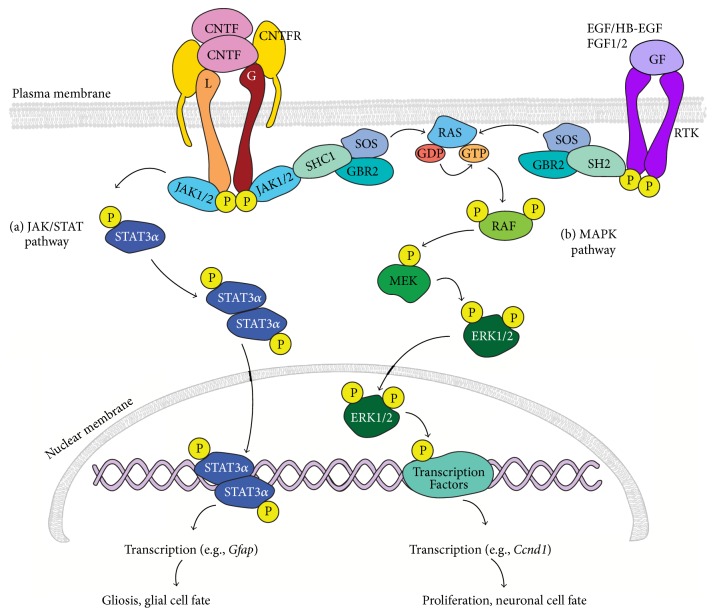
Summary diagram of JAK/STAT and MAPK signal transduction. (a) To activate JAK/STAT signaling, CNTF binding to the GPI linked CNTFR*α* initiates recruitment of LIFR*β* (L) and GP130 (G) to form the hexameric CNTF receptor complex. Recruited LIFR*β* and GP130 are phosphorylated on their cytoplasmic domain by JAK1/2. Activated JAK1/2 phosphorylate STAT3*α*, which forms homodimers that translocate to the nucleus. pSTAT3 homodimers bind to DNA and activate transcription of target genes, such as* Gfap*, to initiate gliosis. (b) The MAPK signaling pathway can be activated downstream of ligand binding to receptor tyrosine-kinases (RTKs) by growth factors including HB-EGF, EGF, FGF1, and FGF2 or by activation of CNTFR by CNTF. In both pathways, adaptor proteins such as GBR2 recruit SOS to the activated receptor, and subsequent activation of SOS leads to phosphorylation of RAS, MEK, and finally ERK1/2. Activated ERK1/2 translocate into the nucleus and phosphorylate several transcription factors involved in cell proliferation, cell survival, and cell differentiation.

Promoting clinically relevant levels of regeneration from Müller glia in the mammalian or human retina will require suppression of gliosis and enhancement of their proliferative and neurogenic responses. Multiple signaling molecules and their downstream signal transduction cascades have been implicated in regulating the injury and regenerative responses in the retina, including notch [85–88], tumor necrosis factor alpha (TNF-*α*) [89], transforming growth factor beta (TGF-*β*) [84, 90–92], insulin [70, 73], midkine (MDKN) [93–95], ciliary neurotrophic factor (CNTF) [75, 92, 96–99], epidermal derived growth factor (EGF) [62, 74, 84, 100], and fibroblast growth factors (FGFs) [62, 75, 101, 102]. Many of these signaling pathways converge on JAK/STAT (Janus kinase/signal transducer and activator of transcription) and MAPK (mitogen-activated protein kinase) signal transduction cascades (Figure 2). Retinal injury stimulates release of a variety of cytokines and mitogens, including EGF, FGF1, FGF2, and CNTF, which have been implicated in various aspects of glial activation and proliferation [103–106]. However, endogenous expression levels of growth factors and cytokines following injury are insufficient to promote significant proliferation of mammalian Müller glia. Injury-induced proliferation can be enhanced by intraocular injection of EGF, FGF1, FGF2, and CNTF, either alone or in various combinations with each other or with insulin [62, 70, 73–75, 92, 98, 101, 107]. All of these factors can activate intracellular signal transduction cascades via JAK/STAT and/or MAPK signaling pathways. Therefore, examination of these signaling pathways and how their activation relates to gliosis and retinal regeneration in fish, birds, and mammals is important to begin to understand the mechanisms contributing to the differential injury responses.

Intracellular signaling through the JAK/STAT pathway is activated by receptor binding of a variety of ligands, including cytokines (e.g., interleukins [108], interferons [109], and CNTF [110]), growth factors (e.g., EGF [111], FGF [112]), and hormones (growth hormone [113], thyrotrophin stimulating hormone [114]). In JAK/STAT signaling, ligand binding to cognate receptors results in phosphorylation of receptor-associated JAK1/2, which causes rapid (within minutes) phosphorylation of STAT3 and a delayed (within hours) phosphorylation of STAT1 [115, 116]. Phosphorylated STAT3 (pSTAT3) and pSTAT1 form homodimers or heterodimers via phosphotyrosyl peptide interaction of their SH2 (Src homology 2) domains, resulting in translocation to the nucleus, binding to DNA at the consensus binding sequence TTCC[C/G]GGAA, and transcription of target genes [116].

CNTF can activate MAPK signaling downstream of its receptor; however activation of MAPK signaling more typically occurs downstream of receptor tyrosine-kinase activation by a variety of ligands, including EGF, FGFs, and insulin. In the MAPK signaling cascade, binding of a signaling ligand to its receptor causes a series of sequential phosphorylation reactions. Each step in the cascade can be performed by multiple proteins, making the cascade both diverse and complex (see reviews in [117, 118]). Briefly, phosphorylation of the cytoplasmic domains of the cytokine receptors causes adaptor molecules to recruit proteins that activate RAS (rat sarcoma oncogene), which phosphorylates RAF (rapidly accelerated fibrosarcoma), which phosphorylates MAPK kinases (including MAPK/ERK kinases 1 and 2; a.k.a. MEK1/MEK2), which phosphorylate MAPKs, including extracellular signal-regulated protein kinase (ERK), c-Jun-N-terminal kinase (JNK), and protein 38 (p38). Phosphorylated MAPK translocates to the nucleus and phosphorylates a variety of transcription factors that activate target gene transcription.

## 6. CNTF Activation of JAK/STAT and Gliosis

Among the factors that can activate JAK/STAT and MAPK signaling, CNTF plays multiple roles in the retinal injury response and particularly in activating the gliotic responses of Müller glia. CNTF expression is upregulated in Müller glia following a variety of retinal injuries in the zebrafish [98], posthatch chicken [75], and mammals [103, 104, 106, 119]. Müller glia of all species express little, if any, GFAP in the absence of injury, neurodegenerative disease, or other insults [67, 120, 121]. However, intraocular injection of CNTF into otherwise uninjured eyes of zebrafish [75], posthatch chickens [92], mice [99], and rats [115] increases GFAP in Müller glia, mimicking a gliotic response [98]. Upregulation of GFAP by CNTF is mediated by STAT3 signaling via direct binding of phosphorylated STAT3 (pSTAT3) dimers to the GFAP promoter [99, 122, 123]. Consistent with a role in gliosis, STAT3 expression also increases in Müller glia following ouabain- or light-induced injury in zebrafish [47, 98], and STAT3 phosphorylation is similarly increased in Müller glia following injury to the avian [75] and mouse retina [124, 125].

## 7. JAK/STAT and MAPK Regulation of Müller Glial Proliferation

In addition to activating JAK/STAT signaling, injury activates MAPK signaling in Müller glia in zebrafish and mice [98, 126]. Various growth factors, including EGF, FGFs, and insulin, activate MAPK signal transduction directly downstream of tyrosine-kinase receptors, whereas CNTF activates MAPK via JAK activation of SHP2/RAS (Figure 2) [98, 127, 128]. Following a penetrating injury, combinatorial treatments of insulin combined with either heparin binding EGF-like growth factor (HB-EGF; an EGF-related but more potent mitogen [129]) or FGF2 increase proliferation of Müller glia in the zebrafish retina, an effect that is reduced by inhibition of MAPK or JAK/STAT [74, 100, 101]. Similarly, in both avian and mouse retinas, NMDA injury increases pERK and pSTAT3 in Müller glia [74, 75, 115]. Proliferation of Müller glia is increased by exogenous HB-EGF, FGF2, insulin, or combinations of CNTF and FGF2, in NMDA injured, but not in uninjured, retinas [75]. Although phosphorylation of ERK1/2 and STAT is increased by these same factors, the ability of CNTF/FGF2 to increase proliferation of Müller glial-derived progenitors in NMDA injured chicken retinas requires activation of JAK/STAT [74, 130]. Similarly, the proliferative effects of CNTF can be blocked at the receptor level by inhibition of the gp-130 coreceptor or postreceptorally by inhibition of either JAK2 phosphorylation or STAT3 dimerization/nuclear translocation [75, 99]. If JAK/STAT signaling mediates both gliosis and proliferation of Müller glia in response of CNTF or injury, a key question is how to promote proliferation of Müller glia-derived stem cells to repopulate areas of retinal degeneration, without also inducing gliosis.

## 8. Müller Glia In Vitro

Despite similarities between fish, birds, and mammals in the Müller glial response to exogenous growth factors, modulation of JAK/STAT and MAPK signaling in the mammalian retina remains insufficient to stimulate clinically relevant regenerative responses. There is evidence that both intrinsic and extrinsic mechanisms contribute to inhibition of the neurogenic/regenerative response of Müller glia in the retinas of higher vertebrates. Although regeneration in the fish retina can occur throughout life, there is an age-associated decline in the proliferative response of Müller glia to retinal injury and exogenous mitogens in rodents [83, 131]. The genetic background in different mouse strains also has significant influence on the ability of Müller glia to proliferate in response to injury and exogenous growth factors, although specific genetic factors have yet to be identified [132]. Since intraocular injection of exogenous factors can promote Müller glial proliferation, it is likely that there are insufficient endogenous levels of known growth factors. In addition, there are likely additional, unidentified factors that are required to activate key signaling pathways or that actively inhibit the proliferative and regenerative capacity in vivo. Consistent with this, isolated mammalian Müller glia show increased proliferation and neurogenic potential in vitro. This may reflect the greater ability to manipulate exogenous factors in vitro, as well as the removal of the Müller glia from any local inhibitory signals. Thus, even though the ultimate goal for using Müller glial-derived progenitors is to stimulate neural regeneration in situ, the use of primary Müller glia and permanent cell lines offers an opportunity to examine mechanisms underlying their proliferative and regenerative responses under more controlled conditions in vitro.

Several Müller glial cell lines have been described: ImM10, conditionally immortalized Müller glia from P10 mouse retinas [61, 133]; MIO-M1, spontaneously immortalized Müller glia from adult human retinas [134]; rMC-1, SV-40 immortalized Müller glia from light-injured rat retinas [135]; TR-MUL5, conditionally immortalized Müller glia from rat [136]; and MU-PH1, conditionally immortalized Müller glia from 2-month-old mice. Direct comparisons are complicated by differences in the age and species of origin, variable culture conditions, and the panels of genes that have been analyzed in different studies. However, in standard culture conditions, all express at least some genes typical of Müller glia in vivo (e.g., vimentin,* Sox2*), as well as nestin, an intermediate filament typically associated with neural stem cells. Only rMC-1 has robust GFAP expression, consistent with its origin from light-injured retinas [135], although one study also reported low levels of GFAP immunoreactivity in MIO-M1 cells [137]. There are concerns that overexpression of oncogenes will fundamentally change the identity and cellular responses of Müller glial cell lines. The conditionally immortalized cell lines, ImM10 (mouse) and TR-MUL5 (rat), contain an inducible, temperature sensitive SV40T-antigen, thereby allowing elimination of oncogene expression under appropriate conditions.

There is a temporal change in cell cycle kinetics of primary Müller glia, which initially proliferate slowly, even in the presence of serum containing medium [133]. After continued culture, primary Müller glia become more highly proliferative, consistent with spontaneous immortalization [133, 134]. Within the first two weeks in culture, Müller glia change morphology and downregulate key genes associated with glial function [e.g., glutamine synthetase (GS), cellular retinaldehyde-binding protein (CRALBP/RLBP1)] [138]. We observed reduced proliferation of ImM10 cells when grown in serum-free medium with nonimmortalizing conditions, although rates of proliferation increased and the differences between immortalizing and nonimmortalizing conditions diminished at higher passage numbers [133]. Thus, careful comparative studies of cultured Müller glia with their in vivo counterparts will be needed for final validation of any identified mechanisms regulating proliferation and neurogenic competence.

**Figure 3:**
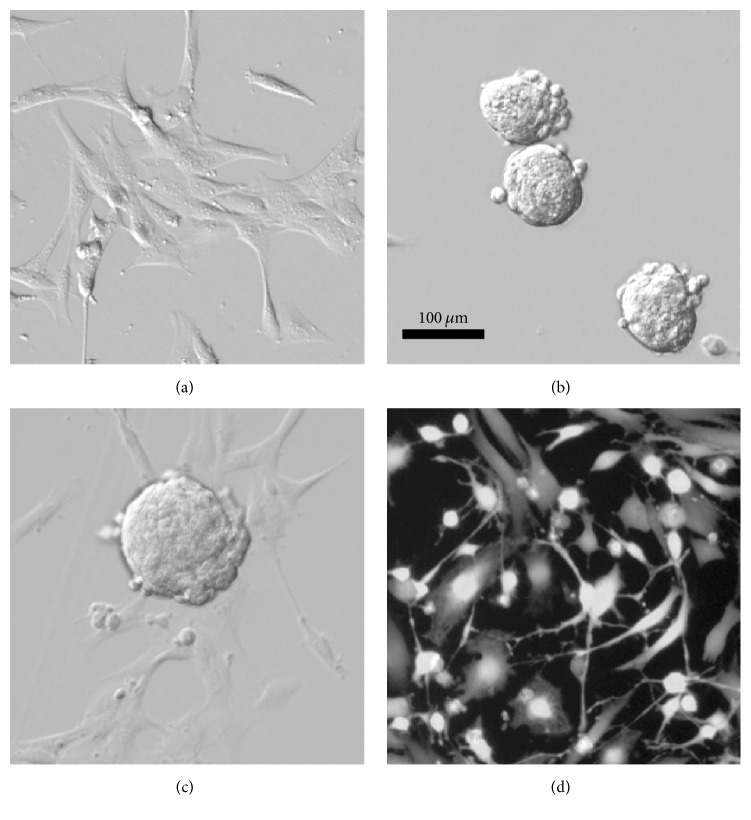
Morphology of mouse Müller glia in growth, neurosphere, and differentiation cultures in vitro. Conditionally immortalized mouse Müller glia (ImM10 cell line) at different stages of in vitro differentiation as previously described [61]. (a) ImM10 cells in growth media (Neurobasal, 2% fetal bovine serum, B27 supplement, and penicillin/streptomycin) under immortalizing conditions (33°C, 50 U/ml interferon gamma) show typical morphology of cultured Müller glia. (b) ImM10 cells following 4 days in sphere forming medium (Neurobasal, B27 supplement, and modified G5 supplement with 20 ng/ml EGF, 20 ng/ml FGF2, and penicillin/streptomycin) in nonimmortalizing conditions to prevent T-antigen expression (39°C, without interferon gamma), showing typical nonadherent neurospheres. (c) Spheres at 1 day following transfer to priming medium (Neurobasal, G5 supplement modified to contain EGF (20 ng/ml) but without FGF2, and penicillin/streptomycin; nonimmortalizing conditions), neurospheres adhere to plate, and cells begin to migrate onto dish. (d) Following priming, ImM10 cells in differentiation medium (Neurobasal, B27, and pen/strep; nonimmortalizing conditions) for 2 days and stained with CalceinAM show variable morphologies and include cells with distinct neuronal morphology (small cell body, multiple thin processes).

Immortalized Müller glia can be induced to generate cells expressing neuronal genes [61, 134, 139–142]. ImM10, MIO-M1, and MU-PH1 generate neurospheres in response to specific growth factors, typically a combination of epidermal derived growth factor (EGF), fibroblast growth factor 2 (FGF2), and/or insulin (Figure 3(b)), and upregulate a variety of genes typical of retinal progenitors, including* Pax6* and nestin [61, 134, 137, 139]. Using a variety of in vitro differentiation protocols, Müller glial-derived progenitors from neurospheres will alter their morphology to resemble cultured neurons, showing condensed nuclei and long, branching neurite-like processes (Figure 3(d)). Redifferentiated human Müller glial-derived progenitors express markers of most retinal cell types and have been shown to respond to light [137]. Transplantation of in vitro differentiated photoreceptors from Müller glial-derived progenitors partially restored light response in a rat model of rapid photoreceptor degeneration, as measured by increases in the a-wave of the electroretinogram (a measure of photoreceptor function) [140]. Transplantation of in vitro differentiated Müller glia into rat retinas, following pharmacological depletion of retinal ganglion cells, partially restored the negative scotopic threshold response of the electroretinogram (an indicator of retinal ganglion cell function) [139]. However, despite the presence of the newly generated cells in their appropriate lamina and evidence of some synapse formation with upstream neurons, the new cells failed to extend axons into the optic nerve or connect to visual centers in the brain. In all differentiation paradigms reported, relatively large numbers of cells continue to express glial genes and retain a glial morphology. Additionally, the number of neurons generated is relatively low, their morphology is inconsistent, and gene expression profiles have yet to demonstrate expression of all genes necessary for specification and functional maturity of individual retinal cell types.

## 9. JAK/STAT and MAPK in Müller Glia In Vitro

There have been few systematic studies of the activity of specific signal transduction cascades in Müller glia in vitro. Since activation of JAK/STAT or MAPK signaling in Müller glia can regulate gliosis and proliferation, we analyzed activation of STAT3 and MAPK pathways in ImM10 cells by western blot. There were no changes in total STAT3 or pSTAT3 in neurosphere or differentiation cultures; and, unexpectedly, despite the presence of both EGF and FGF2 in sphere forming media, there was no change in pERK1/2 in neurospheres [143]. In contrast, pERK1/2 increased in ImM10 cells in differentiation conditions despite the absence of exogenous EGF or FGF2 [143]. However, EGF and FGF2 mRNA levels are upregulated in differentiation cultures of ImM10 cells [61], suggesting that endogenously produced factors contribute to activation of MAPK signaling in these cells.

During neonatal retinal development, CNTF has been proposed to modulate a “molecular switch” that promotes either a glial or neuronal cell fate via concentration dependent activation of STAT3 versus MAPK signaling, respectively [144, 145]. In rat P1 retinal explants and dissociation cultures, low concentrations of CNTF (<50 ng/ml) increase the number of cells expressing neuronal genes via MAPK signaling, whereas high concentrations (100 ng/ml) increase the number of cells expressing glial genes via STAT3 signaling [144]. Inhibition of STAT3 signaling abolishes CNTF's repression of photoreceptor markers in mouse retinal progenitor cell cultures [146, 147], whereas inhibition of MAPK signaling abolishes the increase in the number of cells expressing neuronal markers in dissociation cultures of neonatal rat retina treated with low concentrations of CNTF [144].

To test if low levels of CNTF could promote neurogenesis in differentiation cultures of ImM10 cells, we analyzed gene expression following addition of low concentrations of CNTF (20 ng/ml) [61]. Consistent with our previous study, genes associated with multiple neuronal types were detected in differentiation cultures, including photoreceptors* (Rhodopsin, Opn1sw,* and* Nr2e3)*, retinal ganglion cells* (Sncg, L1Cam)*, and bipolar cells* (Prkca)*. However, addition of CNTF did not alter the overall patterns or levels of gene expression [143]. In addition, GFAP was not detected in differentiation cultures, either with or without CNTF, suggesting that there was no enhancement of gliosis. Surprisingly, CNTF did not change phosphorylation of STAT3 or ERK in ImM10 cells, reflecting the failure of CNTF to activate either JAK/STAT or MAPK signaling. These findings suggest that the ImM10 cells in vitro respond to CNTF differently than Müller glia in vivo. One potential explanation is that the effects of CNTF may require additional cells or factors not present in the cell line. Consistent with this idea, stem cells from dental pulp treated with conditioned media from injured rat retinas upregulated rhodopsin expression in vitro, whereas those treated with conditioned media from purified Müller glia did not [148].

**Figure 4:**
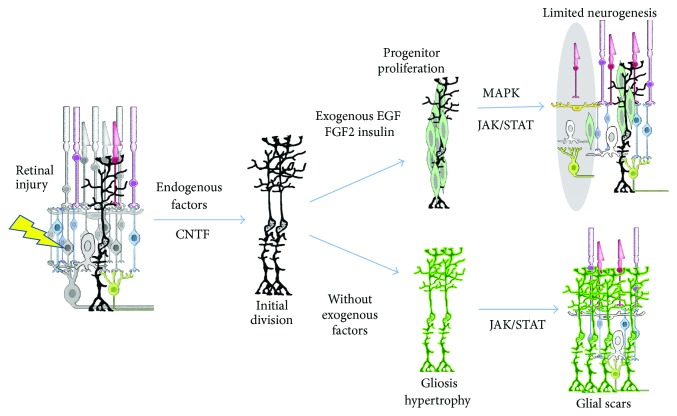
The proposed involvement of CNTF, JAK/STAT, and MAPK signaling in gliosis and neural regeneration by Müller glia in mammalian retina. Retinal injury (lightning bolt) kills retinal cells (gray cells) and stimulates release of growth factors, including CNTF, resulting in limited cell division of Müller glia (MG). In the absence of exogenous growth factors, increased JAK/STAT signaling (lower arrows) in activated MG promotes gliosis (bright green), resulting in glial scars, but neurons are not regenerated. Activation of MAPK and JAK/STAT signaling by exogenous factors, including EGF, FGF2, and insulin (upper arrows), produces proliferative progenitors (light green), which can regenerate some retinal neurons (in gray shaded oval), such as amacrine cells [62] and photoreceptors [63]. Some undifferentiated progenitor cells (light green) persist following resolution of the regenerative response. Even with exogenous factor stimulation, mammalian retinas fail to restore all lost cells.

The JAK/STAT and MAPK signaling pathways are implicated in retinal cell fate choice, proliferation, and gliotic hypertrophy after retinal injury and can be activated by a variety of growth factors including EGF, HB-EGF, FGF2, insulin, and CNTF. The JAK/STAT pathway appears to mediate both beneficial (proliferation) and detrimental (hypertrophy/gliotic) aspects of the injury response in vivo (Figure 4). Our findings that MAPK activation increased in ImM10 cells cultured under conditions that increased neuronal gene expression are consistent with a role for MAPK signaling in mediating a neuronal cell fate over glial fate choice. In mammalian retinal injury, the gliotic response predominates over the regenerative response, although the regenerative response can be enhanced with the addition of exogenous growth factors and cytokines that activate JAK/STAT and MAPK signaling. Unfortunately, this enhancement is still insufficient to make regeneration predominate over gliosis.

## 10. Other Mechanisms

Although activation of JAK/STAT and MAPK signaling by CNTF, EGF, and FGFs can promote proliferation and the glial/neurogenic switch, these signal transduction pathways do not act in isolation. Rather, they function within the context of a wide variety of other cellular mechanisms that contribute to the retinal injury response and regeneration in various contexts. Extensive discussion of all the cellular mechanisms regulating the injury and regenerative responses of Müller glia is beyond the scope of this article; several recent reviews can provide more information [22, 64, 149–154]. In addition to its roles in regulating gliosis and cell fate specification, CNTF is also implicated in neuroprotection and axonal outgrowth through activation of the MAPK, JAK/STAT, and/or PI3K (phosphatidylinositol-3 kinase) pathway [155–159]. Other pathways that are important in regulating cell cycle reentry and exit, gliosis, neurogenesis, and differentiation of Müller glia following retinal injury include the following: notch [85, 87, 160] and WNT signaling [161, 162]; activation of a variety of signal transduction cascades by TNF-*α* [87, 89], TGF-*β* [163], and BMP/SMADs [84, 91, 164]; regulation of cell cycle by* ccnd1* and* p27*(*kip1*) [83, 85, 165]; microRNAs [78, 166–168]; and the effects of transcriptional regulators that regulate retinal development, such as the proneural gene* Ascl1a* [79, 142, 162] and the neurogenic gene* Atoh7* [80, 81, 141]. Thus, expanding our understanding of how multiple pathways integrate to regulate the injury and regenerative responses of Müller glial will be important for continued progress in the field.

Analysis of retinal regeneration in fish has identified a variety of signaling molecules and their downstream signal transduction cascades that have shown promise for enhancing regenerative responses of Müller glia and warrant continued study. One intriguing, yet understudied, molecule that plays a role in retinal neurogenesis and regeneration in zebrafish is midkine (MDKN) [169]. MDKN is a heparin binding protein that interacts, either directly or indirectly, with a number of receptors, including ALK, LRP, notch, and protein tyrosine phosphatase-zeta (PTP-*ξ*), to modulate downstream signal transduction cascades [94, 169–174]. Binding of MDKN, or the structurally related pleiotrophin, to PTP-*ξ* blocks its phosphatase activity, resulting in increased phosphorylation of a variety of tyrosine-kinase receptors and their substrates and potentiating downstream signal transduction cascades [94]. In zebrafish, MDKNa/MDKNb are expressed in both retinal progenitors and Müller glia and are upregulated following retinal injury [93, 95]. Morpholino inhibition of MDKNa reduces proliferation of Müller glial-derived progenitors and limits regeneration of rod photoreceptors following light damage in zebrafish [93]. Much less is known about MDKN in the mammalian retina, although it is neuroprotective for rod photoreceptors following light damage in mice [175] and rats [176]. We found that* Mdkn* mRNA is upregulated in ImM10 cells under differentiation condition in vitro [61]. Given the pleiotropic effects of MDKN and its potential to modulate multiple signal transduction cascades that impact the proliferative and neurogenic responses of Müller glia, it would be interesting to assess combinatorial effects of MDKN and growth factor stimulation of JAK/STAT, MAPK, or other signal transduction cascades on Müller glial proliferation and retinal regeneration.

## 11. Conclusions

Müller glia are particularly appealing as a cellular source for retinal regeneration because they are intrinsic to the retina and offer the potential to regenerate neurons in situ, without transplantation. Despite a growing body of research showing the neurogenic potential of Müller glia in the mammalian retina, a level of regenerative response sufficient for potential clinical applications has yet to be achieved. Given the overall modest outcomes to date, cultured Müller glia seem unlikely to provide a clinically relevant path for generating sufficiently large numbers of retinal progenitors for transplantation. Successful regenerative strategies using transplantation are more likely to build on the ongoing progress in generating retinal progenitors and neurons from other sources, such as induced pluripotent stem cells [3–6, 15, 177–184]. Therefore, the importance of studies to promote in vitro differentiation of Müller glia lies in the ability to manipulate the cellular environment and to dissect cellular mechanisms that regulate their regenerative responses. Nevertheless, there is clear evidence that Müller glia change patterns of proliferation and gene expression as they adapt to culture conditions and that the ability of Müller glia to respond to some ligands and activate key signaling pathways is different in vitro and in vivo. This raises questions about whether in vitro assays will recapitulate all aspects of the in vivo glial injury response and how in vitro findings will translate into promoting regeneration in vivo. Thus, caution is warranted in interpreting results obtained using Müller glial cell lines. Research to promote clinically relevant levels of retinal regeneration from mammalian Müller glia will benefit from the use of more complex model systems and such as ex vivo retinal explants and three-dimensional substrates and the inclusion of multiple retinal cell types to better model the retinal environment.
